# Hypertrophic pachymeningitis in ANCA-associated vasculitis: a cross-sectional and multi-institutional study in Japan (J-CANVAS)

**DOI:** 10.1186/s13075-022-02898-4

**Published:** 2022-08-23

**Authors:** Yasuhiro Shimojima, Dai Kishida, Takanori Ichikawa, Takashi Kida, Nobuyuki Yajima, Satoshi Omura, Daiki Nakagomi, Yoshiyuki Abe, Masatoshi Kadoya, Naoho Takizawa, Atsushi Nomura, Yuji Kukida, Naoya Kondo, Yasuhiko Yamano, Takuya Yanagida, Koji Endo, Shintaro Hirata, Kiyoshi Matsui, Tohru Takeuchi, Kunihiro Ichinose, Masaru Kato, Ryo Yanai, Yusuke Matsuo, Ryo Nishioka, Ryota Okazaki, Tomoaki Takata, Takafumi Ito, Mayuko Moriyama, Ayuko Takatani, Yoshia Miyawaki, Toshiko Ito-Ihara, Takashi Kawaguchi, Yutaka Kawahito, Yoshiki Sekijima

**Affiliations:** 1 Department of Medicine (Neurology and Rheumatology), Shinshu University School of Medicine, 3-1-1 Asahi, Matsumoto, 390-8621 Japan; 2 Inflammation and Immunology, Graduate School of Medical Science, Kyoto Prefectural University of Medicine, Kyoto, Japan; 3 Division of Rheumatology, Department of Medicine, Showa University School of Medicine, Tokyo, Japan; 4 Department of Rheumatology, University of Yamanashi Hospital, Yamanashi, Japan; 5 Department of Internal Medicine and Rheumatology, Juntendo University, Tokyo, Japan; 6 Center for Rheumatic Disease, Japanese Red Cross Society Kyoto Daiichi Hospital, Kyoto, Japan; 7 Department of Rheumatology, Chubu Rosai Hospital, Nagoya, Japan; 8 Immuno-Rheumatology Center, St. Luke’s International Hospital, Tokyo, Japan; 9 Department of Rheumatology, Japanese Red Cross Society Kyoto Daini Hospital, Kyoto, Japan; 10 Department of Nephrology, Kyoto Katsura Hospital, Kyoto, Japan; 11 Department of Respiratory Medicine and Allergy, Tosei General Hospital, Seto, Aichi Japan; 12 Department of Hematology and Rheumatology, Kagoshima University Hospital, Kagoshima, Japan; 13 Department of Pharmacoepidemiology, Graduate School of Medicine and Public Health, Kyoto University, Kyoto, Japan; 14 Department of General Internal Medicine, Tottori Prefectural Central Hospital, Tottori, Japan; 15 Department of Clinical Immunology and Rheumatology, Hiroshima University Hospital, Hiroshima, Japan; 16 Department of Diabetes, Endocrinology and Clinical Immunology, Hyogo Medical University School of Medicine, Nishinomiya, Hyogo Japan; 17 Department of Internal Medicine (IV), Osaka Medical and Pharmaceutical University, Osaka, Japan; 18 Department of Immunology and Rheumatology, Division of Advanced Preventive Medical Sciences, Nagasaki University Graduate School of Biomedical Sciences, Nagasaki, Japan; 19 Department of Rheumatology, Endocrinology and Nephrology, Graduate School of Medicine, Hokkaido University, Sapporo, Japan; 20 Department of Rheumatology, Tokyo Kyosai Hospital, Tokyo, Japan; 21 Department of Rheumatology, Graduate School of Medical and Dental Sciences, Tokyo Medical and Dental University, Tokyo, Japan; 22 Department of Rheumatology, Graduate School of Medical Science, Kanazawa University, Kanazawa, Japan; 23 Division of Respiratory Medicine and Rheumatology, Department of Multidisciplinary Internal Medicine, Faculty of Medicine, Tottori University, Yonago, Japan; 24 Division of Gastroenterology and Nephrology, Tottori University, Yonago, Japan; 25 Division of Nephrology, Shimane University Hospital, Izumo, Shimane Japan; 26 Department of Rheumatology, Shimane University Faculty of Medicine, Izumo, Shimane Japan; 27 Rheumatic Disease Center, Sasebo Chuo Hospital, Nagasaki, Japan; 28 Department of Nephrology, Rheumatology, Endocrinology and Metabolism, Okayama University Graduate School of Medicine, Dentistry and Pharmaceutical Sciences, Okayama, Japan; 29 The Clinical and Translational Research Center, University Hospital, Kyoto Prefectural University of Medicine, Kyoto, Japan; 30 Department of Practical Pharmacy, Tokyo University of Pharmacy and Life Sciences, Tokyo, Japan

**Keywords:** Hypertrophic pachymeningitis, Antineutrophil cytoplasmic antibody, ANCA-associated vasculitis, Granulomatosis with polyangiitis, Ear, nose, and throat, Mucous membranes/eyes, Sudden visual loss, Conductive hearing loss

## Abstract

**Background:**

This study investigated the characteristics of hypertrophic pachymeningitis (HP) in antineutrophil cytoplasmic antibody (ANCA)-associated vasculitis (AAV), using information from a multicenter study in Japan.

**Methods:**

We analyzed the clinical information of 663 Asian patients with AAV (total AAV), including 558 patients with newly diagnosed AAV and 105 with relapsed AAV. Clinical findings were compared between patients with and without HP. To elucidate the relevant manifestations for HP development, multivariable logistic regression analyses were additionally performed.

**Results:**

Of the patients with AAV (mean age, 70.2 ± 13.5 years), HP was noted in 30 (4.52%), including 20 (3.58%) with newly diagnosed AAV and 10 (9.52%) with relapsed AAV. Granulomatosis with polyangiitis (GPA) was classified in 50% of patients with HP. A higher prevalence of GPA was significantly observed in patients with HP than in those without HP in total AAV and newly diagnosed AAV (*p* < 0.001). In newly diagnosed AAV, serum proteinase 3 (PR3)-ANCA positivity was significantly higher in patients with HP than in those without HP (*p* = 0.030). Patients with HP significantly had ear, nose, and throat (ENT) (odds ratio [OR] 1.48, 95% confidence interval [CI] 1.03–2.14, *p* = 0.033) and mucous membrane/eye manifestations (OR 5.99, 95% CI 2.59–13.86, *p* < 0.0001) in total AAV. Moreover, they significantly had conductive hearing loss (OR 11.6, 95% CI 4.51–29.57, *p* < 0.0001) and sudden visual loss (OR 20.9, 95% CI 5.24–85.03, *p* < 0.0001).

**Conclusion:**

GPA was predominantly observed in patients with HP. Furthermore, in newly diagnosed AAV, patients with HP showed significantly higher PR3-ANCA positivity than those without HP. The ear and eye manifestations may be implicated in HP development.

**Supplementary Information:**

The online version contains supplementary material available at 10.1186/s13075-022-02898-4.

## Introduction

Antineutrophil cytoplasmic antibody (ANCA)-associated vasculitis (AAV), which systemically affects small- and medium-sized vessels, involves visceral impairments that can lead to life-threatening complications. AAV can be categorized into two different types based on the target antigen for ANCA, which is implicated in the pathogenesis of AAV, including myeloperoxidase (MPO) and proteinase 3 (PR3) [1]. Furthermore, microscopic polyangiitis (MPA), granulomatosis with polyangiitis (GPA), and eosinophilic granulomatosis with polyangiitis (EGPA) are common classifications of AAV [1–3]. All categories of AAV generally involve neurological manifestations, although those in the peripheral nervous system are more common than those in the central nervous system (CNS) [4, 5]. However, CNS manifestations, such as intracranial ischemia or hemorrhage attributed to vasculitis, parenchymal lesions, and encephalopathy [6, 7], are broadly involved in < 15 to > 50% of patients with AAV [4–6]. Notably, hypertrophic pachymeningitis (HP) can also develop as a CNS manifestation in AAV [6–8]. HP is characterized as an inflammatory disorder indicating intracranial or spinal thickening of the dura mater, and its pathology includes inflammatory cell infiltration and interstitial tissue fibrosis [9, 10]. HP development is secondarily ascribable to CNS infections, neoplasms, or autoinflammatory disorders; meanwhile, ANCA is more frequently implicated in the pathogenesis of immune-mediated HP [9–12]. HP could develop as the first clinical episode of AAV, whereas approximately 15% of patients with HP, even with serum positivity for ANCA, cannot be classified as having definite AAV [10, 12], suggesting that HP could be a CNS-limited type of AAV [8, 10]. Moreover, patients with immune-mediated HP showing serum positivity for ANCA without a definite classification of AAV have been comprehensively categorized as ANCA-associated HP [10–16]. Given these unclassifiable categories of immune-mediated HP with ANCA positivity, it is necessary to specifically understand the clinical characteristics of HP developing in AAV. Several studies focusing on HP in AAV have been reported to date [8, 17–20]. To the best of our knowledge, these previous studies were single-institution studies. Furthermore, their analyses were performed in a small number of patients, in whom newly diagnosed AAV and relapsed AAV were simultaneously included, because HP is a rare neurological disorder. Of them, some studies described that the ear, nose, and throat (ENT) or eye manifestations were frequently shown in patients with HP [8, 10, 17–19]; however, it is necessary to validate the relationship between these suggestive manifestations and the development of HP by analyzing a large number of patients with AAV.

In this study, we clarified the epidemiological and clinical characteristics of AAV by comparing patients with HP to those without HP. The analyses were performed using the information from a multicenter study in Japan (Japan Collaborative Registry of ANCA-Associated Vasculitis (J-CANVAS)), ultimately allowing for demonstrating the results acquired from a larger number of patients than those in the previous studies.

## Methods

### Patients and database

This study was performed using the clinical information of 663 Asian patients with AAV who were enrolled in the J-CANVAS from 26 divisions in 24 Japanese institutions. The enrolled patients had newly diagnosed AAV or relapsed AAV between January 2017 and June 2020. All patients were aged > 20 years and classified as having MPA, GPA, or EGPA based on the 2012 International Chapel Hill Consensus Conference on the Nomenclature of Vasculitides [2] and the European Medicines Agency algorithm [3]. The information regarding the diagnosis or relapse of AAV, including epidemiological, laboratory, and radiological findings and clinical manifestations related to AAV based on the Birmingham Vasculitis Activity Score version 3 (BVAS) [21], was retrospectively extracted from the medical records of each institution. All extracted data were collected using an electric data capture system, Viedoc (PCG Solutions, Uppsala, Sweden). HP was defined as radiological findings showing thickening of the cranial or spinal dura mater on magnetic resonance imaging (MRI).

### Study design

In this cross-sectional study, epidemiological, clinical, and laboratory findings, including MPO- and PR3-ANCA, were compared between patients with and without HP. These parameters were evaluated separately in all the enrolled patients, patients with newly diagnosed AAV, and those with relapsed AAV. The overall disease activity, whose scale is determined to be between 0 and 63, and the incidence of manifestations related to AAV were evaluated according to the BVAS [21]. In addition, the scores of each manifestation were also evaluated based on the BVAS: (1) general (scale range, 0–3), (2) cutaneous (0–6), (3) mucous membranes/eyes (0–6), (4) ENT (0–6), (5) chest (0–6), (6) cardiovascular (0–6), (7) abdominal (0–9), (8) renal (0–12), and (9) nervous system (0–9). To elucidate the relevant manifestations for HP development, multivariable analyses were performed on all the enrolled total patients.

This study was conducted based on the ethical standards of the Helsinki Declaration and the ethical guideline for epidemiologic research of the Ministry of Health, Labour and Welfare of Japan. This study was approved by the local institutional review boards (IRBs) of all participating institutions including the local ethics committee of Shinshu University (the approval number: 5126/5390). The IRBs waived written informed consent in this noninvasive and observational study based on the abovementioned ethical guidelines. Accordingly, all patients received information on this study through an opt-out method and thus had the opportunity to decline their participation.

### Statistical analyses

All data are presented as the mean ± standard deviation (SD), and two-sided *p*-values < 0.05 were considered statistically significant. The Mann-Whitney *U* test and Fisher’s exact probability test were used to compare the patients with and without HP. Multivariable logistic regression analyses were used to evaluate the relationships between relevant manifestations, including mucous membrane/eye and ENT manifestations which were determined based on previous studies [8, 10, 17–19], and HP development by estimating a regression coefficient with a two-sided 95% confidence interval (CI). They were adjusted for age, sex, and lung and renal manifestations as potential confounding variables based on previous studies and clinical perspectives. Statistical analyses were performed using the JMP 14.3.0 software (SAS Institute Inc., Cary, NC).

## Results

### Epidemiologic and laboratory findings in patients with HP

The mean age and male-to-female ratio were 70.2 ± 13.5 years and 1:1.22, respectively, in 663 patients (total AAV), including 558 with newly diagnosed AAV (mean age, 70.8 ± 13.2 years; male-to-female ratio, 1:1.20) and 105 with relapsed AAV (66.8 ± 14.5 years; 1:1.39). The mean disease duration of patients with relapsed AAV was 5.7 ± 5.6 years. Of total AAV, HP was observed in 30 (4.52%) patients (Table 1). They were equally classified as having GPA or MPA (*n* = 15 [50%] each). A significantly higher classification of GPA was shown in patients with HP than in those without HP (*p* = 0.0003), although that of MPA was not significantly different (*p* = 0.341). EGPA was not classified in patients with HP, whereas 126 (20%) patients without HP were classified as having EGPA (*p* = 0.003). Patients with HP had higher serum positivity for MPO-ANCA (*n* = 23 [77%]) than for PR3-ANCA (*n* = 6 [20%]), while their frequencies were not significantly different between patients with and without HP. Negative ANCA results were observed in one (3%) patient with HP and 82 (13%) without HP (*p* = 0.159). Laboratory data demonstrated significantly lower number of white blood cells and lymphocytes (Lym) (*p* = 0.009, *p* = 0.020, respectively) in patients with HP, whereas higher serum creatinine (Cre) levels and lower estimated glomerular filtration rate (eGFR) were significantly more frequent in those without HP (*p* = 0.005, *p* = 0.0005, respectively).

**Table 1:** Demographic and laboratory findings between AAV patients with and without HP

	Total AAV			Newly diagnosed AAV			Relapsed AAV		
	HP (*n* = 30)	Non-HP (*n* = 633)	*p*-values	HP (*n* = 20)	Non-HP (*n* = 538)	*p*-values	HP (*n* = 10)	Non-HP (*n* = 95)	*p*-values
Characteristics									
Age, years	71.1 ± 8.0	70.2 ± 13.7	0.509	70.8 ± 8.5	70.9 ± 13.4	0.349	71.9 ± 7.5	66.3 ± 15.0	0.425
Sex, male/female, *n* (%)	18/12	280/353	0.095	12/8	280/353	0.177	6/4	38/57	0.315
AAV classification, *n* (%)									
GPA	15 (50)	126 (20)	0.0003	10 (50)	89 (17)	0.0007	5 (50)	37 (39)	0.516
MPA	15 (50)	309 (60)	0.341	10 (50)	345 (64)	0.238	5 (50)	36 (38)	0.507
EGPA	0	126 (20)	0.003	0	104 (19)	0.345	0	22 (23)	0.116
Type of ANCA, *n* (%)									
PR3	6 (20)	78 (12)	0.254	5 (25)	47 (9)	0.030	1 (10)	31 (32)	0.277
MPO	23 (77)	473 (75)	0.999	14 (70)	421 (78)	0.409	9 (90)	52 (55)	0.042
Seronegative	1 (3)	82 (13)	0.159	1 (5)	70 (13)	0.494	0	12 (13)	0.599
Laboratory data									
White blood cells, /μL	9618 ± 4124	12398 ± 6425	0.009	10510 ± 4628	12729 ± 6533	0.129	7834 ± 2069	10528 ± 5435	0.084
Neutrophils, /μL	8017 ± 4189	8456 ± 4487	0.586	8635 ± 4889	8569 ± 4588	0.936	6842 ± 2102	7830 ± 3848	0.523
Lymphocytes, /μL	1045 ± 630	1326 ± 748	0.020	1269 ± 632	1357 ± 756	0.826	587 ± 310	1153 ± 679	0.002
C-reactive protein, mg/dL	5.22 ± 5.59	7.12 ± 6.27	0.505	7.35 ± 5.70	7.73 ± 6.33	0.892	3.97 ± 4.86	3.68 ± 4.70	0.674
Serum creatinine, mg/dL	0.79 ± 0.36	1.44 ± 1.55	0.005	0.78 ± 0.33	1.45 ± 1.57	0.019	0.82 ± 0.44	1.39 ± 1.43	0.111
eGFR	75.7 ± 24.8	57.2 ± 31.9	0.0005	75.8 ± 23.0	57.1 ± 32.1	0.004	76.5 ± 29.5	57.8 ± 31.3	0.077

Values are expressed as the mean ± SD. Statistical significance was set at *p*-value < 0.05

*ANCA* antineutrophil cytoplasmic antibody, *AAV* ANCA-associated vasculitis, *GPA* eosinophilic granulomatosis with polyangiitis, *eGFR* estimated glomerular filtration rate, *GPA* granulomatosis with polyangiitis, *HP* hypertrophic pachymeningitis, *MPA* microscopic polyangiitis

In newly diagnosed AAV, 20 (3.58%) patients had HP (Table 1). GPA and MPA were classified equally in 10 (50%) patients with HP. The GPA classification was significantly higher in patients with HP than in those without HP (*p* = 0.0007). The serum positivity for MPO-ANCA was higher (*n* = 14 [70%]) than that for PR3-ANCA (*n* = 5 [25%]) in patients with HP. Nevertheless, a higher serum positivity for PR3-ANCA was significantly observed in patients with HP than in those without HP (*p* = 0.030). Patients without HP had significantly higher Cre levels and lower eGFR than those with HP (*p* = 0.019, *p* = 0.004, respectively).

In relapsed AAV, HP was demonstrated in 10 (9.52%) patients (Table 1), which was a significantly higher frequency than that in newly diagnosed AAV (*p* = 0.017) (Additional file 1: Table S1). Of the ten patients with HP, five (50%) were equally classified as having GPA and MPA. There were no significant differences in the AAV classification between patients with and without HP. Meanwhile, patients without HP showed a significantly higher prevalence of GPA and lower prevalence of MPA in relapsed AAV than in newly diagnosed AAV (*p* < 0.0001, *p* < 0.0001, respectively). Patients with HP had higher serum positivity for MPO-ANCA (*n* = 9 [90%]) than for PR3-ANCA (*n* = 1 [10%]), and MPO-ANCA serum positivity was significantly higher in patients with HP than in those without HP (*p* = 0.042). Laboratory findings revealed significantly lower Lym counts in patients with HP than in those without HP (*p* = 0.002).

### Clinical characteristics based on BVAS in patients with HP

The incidence and scores of mucous membrane/eye and ENT manifestations were significantly higher in patients with HP than in those without HP in total AAV (incidence: *p* < 0.0001, *p* = 0.001, respectively) (scores: *p* < 0.0001, *p* < 0.0001, respectively) (Table 2). In the analyses of newly diagnosed AAV, these manifestations also had a significantly higher incidence and scores in patients with HP than in those without HP (incidence: *p* = 0.007, *p* = 0.0002, respectively) (scores: *p* = 0.013, *p* < 0.0001, respectively). In relapsed AAV, the incidence and scores of mucous membrane/eye manifestation were significantly higher in patients with HP than in those without HP (*p* = 0.002, *p* = 0.0001, respectively), whereas those of ENT manifestations were not significantly different. Meanwhile, significantly higher rates of complications of “sudden visual loss” and “conductive hearing loss,” which are classified as mucous membrane/eye and ENT manifestations, respectively [21], were demonstrated in patients with HP than in those without HP in each analysis of total AAV (*p* < 0.0001, *p* < 0.0001, respectively), newly diagnosed AAV (*p* = 0.012, *p* < 0.0001, respectively), and relapsed AAV (*p* = 0.001, *p* = 0.044, respectively) (Table 3). Conversely, the incidence of cutaneous and renal manifestations was significantly lower in patients with HP than in those without HP in total AAV (*p* = 0.042, *p* = 0.003, respectively). The scores of renal manifestations were also significantly lower in patients with HP than in those without HP in the analyses of total AAV and newly diagnosed AAV (*p* = 0.0008, *p* = 0.002, respectively). In relapsed AAV, the incidence and scores of renal manifestations were not significantly different between patients with and without HP; however, those in patients without HP were significantly lower in relapsed AAV than in newly diagnosed AAV (*p* < 0.0001, *p* < 0.0001, respectively). Moreover, incidence and scores of renal manifestations were not significantly different between patients with HP in newly diagnosed AAV and those in relapsed AAV (Additional file 1: Table S1).

**Table 2:** Incidence and scores of manifestations related to AAV between AAV patients with and without HP

	Total AAV			Newly diagnosed AAV			Relapsed AAV		
	HP (*n* = 30)	Non-HP (*n* = 633)	*p*-values	HP (*n* = 20)	Non-HP (*n* = 538)	*p*-values	HP (*n* = 10)	Non-HP (*n* = 95)	*p*-values
**BVAS, total**									
Total score	13.4 ± 5.56	14.9 ± 7.11	0.364	14.0 ± 5.40	15.7 ± 7.01	0.362	12.3 ± 5.98	10.6 ± 6.02	0.390
**Manifestation**									
**Incidence**, number (%)									
General	13 (43)	367 (58)	0.132	11 (55)	335 (62)	0.640	2 (20)	32 (34)	0.494
Cutaneous	2 (7)	149 (24)	0.042	2 (10)	136 (25)	0.184	0	13 (14)	0.357
Mucous membranes/eyes	11 (37)	55 (8)	< 0.0001	6 (30)	47 (9)	0.007	5 (50)	8 (8)	0.002
ENT	16 (53)	157 (25)	0.001	13 (65)	135 (25)	0.0002	3 (30)	22 (23)	0.698
Chest	10 (33)	272 (46)	0.194	7 (35)	255 (47)	0.363	3 (30)	35 (37)	0.745
Cardiovascular	0	46 (7)	0.257	0	42 (8)	0.388	0	4 (4)	0.999
Abdominal	0	24 (4)	0.619	0	21 (4)	0.999	0	3 (3)	0.999
Renal	12 (40)	427 (67)	0.003	10 (50)	381 (71)	0.077	2 (20)	46 (48)	0.106
Nervous system	25 (83)	232 (37)	< 0.0001	18 (90)	195 (36)	< 0.0001	7 (70)	37 (39)	0.090
**Score**^a^									
General	1.00 ± 1.25	1.34 ± 1.28	0.153	1.35 ± 1.31	1.47 ± 1.28	0.612	0.40 ± 0.84	0.63 ± 0.99	0.421
Cutaneous	0.13 ± 0.51	0.58 ± 1.17	0.029	0.20 ± 0.62	0.63 ± 1.21	0.111	0	0.29 ± 0.89	0.215
Mucous membranes/eyes	1.62 ± 2.40	0.30 ± 1.13	< 0.0001	1.05 ± 1.93	0.29 ± 1.07	0.013	2.60 ± 2.99	0.40 ± 1.42	0.0001
ENT	2.52 ± 2.62	0.95 ± 1.88	< 0.0001	3.05 ± 2.56	0.95 ± 1.87	< 0.0001	1.50 ± 2.55	0.94 ± 1.96	0.529
Chest	1.50 ± 2.29	2.07 ± 2.41	0.184	1.45 ± 2.16	2.13 ± 2.41	0.191	1.60 ± 2.63	1.69 ± 2.38	0.834
Cardiovascular	0	0.40 ± 1.46	0.126	0	0.43 ± 1.52	0.194	0	0.22 ± 1.09	0.510
Abdominal	0	0.30 ± 1.58	0.278	0	0.32 ± 1.63	0.368	0	0.22 ± 1.25	0.571
Renal	3.13 ± 4.45	6.37 ± 5.10	0.0008	3.60 ± 4.38	6.85 ± 5.07	0.005	2.20 ± 4.66	3.66 ± 4.46	0.191
Nervous system	3.53 ± 3.22	2.61 ± 3.73	0.0008	3.30 ± 3.16	2.64 ± 3.76	0.002	4.00 ± 3.46	2.48 ± 3.54	0.114

Statistical significance was set at a *p*-value < 0.05. Values are expressed as mean ± SD

*ANCA* antineutrophil cytoplasmic antibody, *AAV* ANCA-associated vasculitis, *BVAS* Birmingham Vasculitis Activity Score version 3, *ENT* eye, nose, and throat, *HP* hypertrophic pachymeningitis

^a^Scored for each manifestation item based on the BVAS [21]

**Table 3:** Comparison of clinical items in mucous membrane/eye and ENT manifestation between patients with and without HP

	Total AAV			Newly diagnosed AAV			Relapsed AAV		
	HP (*n* = 30)	Non-HP (*n* = 633)	*p*-values	HP (*n* = 20)	Non-HP (*n* = 538)	*p*-values	HP (*n* = 10)	Non-HP (*n* = 95)	*p*-values
**Manifestation**									
**Mucous membranes/eyes**, number (%)									
Mouth ulcers	0	9 (1.4)	0.999	0	9 (1.7)	–	–	0	–
Genital ulcers	0	0	–	0	0	–	–	0	–
Adnexal inflammation	0	0	–	0	0	–	–	0	–
Significant proptosis	0	5 (0.8)	1	0	2 (0.4)	1	0	3 (3.2)	0.999
Scleritis/episcleritis	5 (17)	29 (4.6)	0.015	3 (15)	26 (4.8)	0.079	2 (20)	3 (3.2)	0.070
Conjunctivitis/blepharitis/keratitis	0	7 (1.1)	0.999	0	7 (1.3)	0.999	0	0	–
Blurred vision	2 (6.7)	10 (1.6)	0.099	1 (5)	8 (1.5)	0.282	1 (10)	2 (2.1)	0.262
Sudden visual loss	6 (20)	6 (0.9)	< 0.0001	2 (10)	3 (0.5)	0.012	4 (40)	3 (3.2)	0.001
Uveitis	0	4 (0.6)	1	0	5 (0.9)	0.999	0	0	–
Retinal changes (vasculitis, thrombosis/exudate/hemorrhage)	0	5 (0.8)	0.999	0	3 (0.5)	0.999	0	1 (1.1)	1
**ENT**, number (%)									
Bloody nasal discharge/crusts/ulcers/granulomata	2 (6.7)	46 (7.2)	0.999	1 (5)	38 (7.1)	0.999	1 (10)	8 (8)	0.999
Paranasal sinus involvement	7 (23)	108 (17)	0.334	5 (17)	76 (17)	0.371	2 (20)	15 (16)	0.663
Subglottic stenosis	0	1 (0.2)	1	0	0	–	0	1 (1.1)	1
Conductive hearing loss	11 (37)	42 (6.6)	< 0.0001	9 (45)	40 (7.4)	< 0.0001	2 (20)	2 (2.1)	0.044
Sensorineural hearing loss	7 (23)	25 (3.9)	0.0003	5 (25)	20 (3.7)	0.001	2 (20)	5 (5.3)	0.133

Statistical significance was set at *p*-value < 0.05

*ANCA* antineutrophil cytoplasmic antibody, *AAV* ANCA-associated vasculitis, *ENT* ear, nose, and throat, *HP* hypertrophic pachymeningitis

**Fig. 1:**
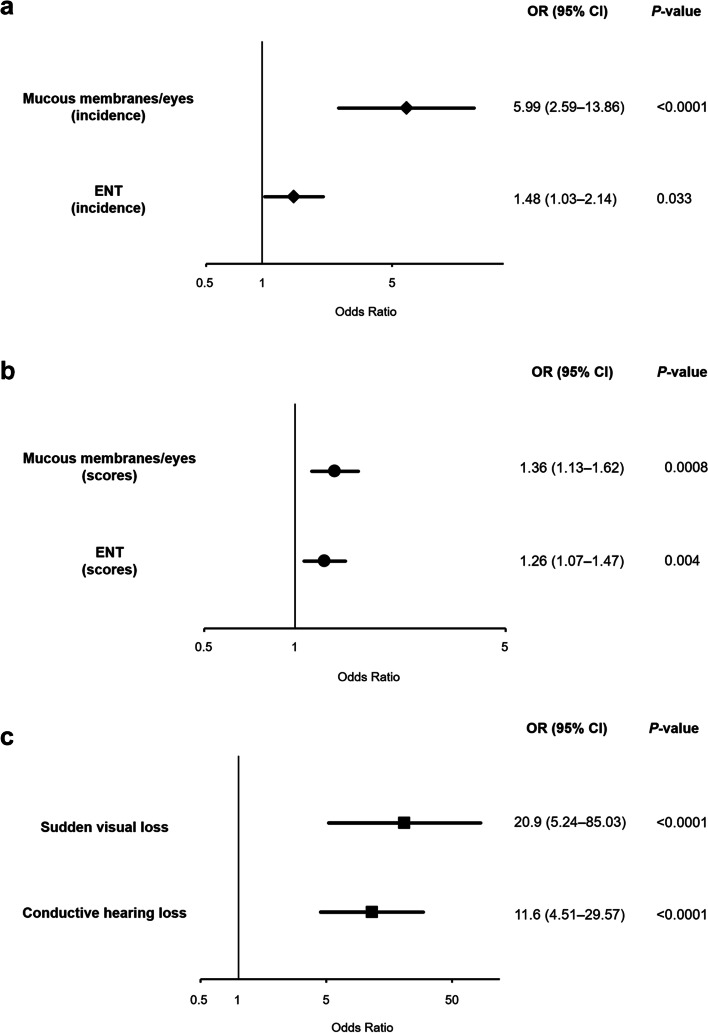
Multivariable logistic analyses of significant manifestations related to antineutrophil cytoplasmic antibody (ANCA)-associated vasculitis in patients with hypertrophic pachymeningitis after adjustment for potential confounding variables. Incidence (**a**) and scores (**b**) of the mucous membrane/eye and ear, nose, and throat (ENT) manifestations and incidence of sudden visual loss and conductive hearing loss (**c**) were analyzed. OR, odds ratio; CI, confidence interval

These results suggest that mucous membrane/eye and ENT manifestations may be implicated in the development of HP. Moreover, previous studies indicated the frequent existence of these two manifestations in patients with HP in AAV [8, 10, 17–19]. Next, the relationships between these two manifestations and HP development were evaluated in total AAV. In multivariable logistic regression analyses after adjustment for potential confounding variables including age, sex, and lung and renal manifestations, HP was significantly associated with an incidence of mucous membrane/eye (odds ratio [OR] 5.99, 95% CI 2.59 to 13.86, *p* < 0.0001) and ENT (OR 1.48, CI 1.03 to 2.14, *p* = 0.033) manifestations (Fig. 1a). Patients with HP also significantly demonstrated the association with scores for membrane/eye (OR 1.36, 95% CI 1.13–1.62, *p* = 0.0008) and ENT (OR 1.26, 95% CI 1.07–1.47, *p* = 0.004) manifestations (Fig. 1b). In addition, sudden visual loss and conductive hearing loss were significantly identified in patients with HP (OR 20.9, 95% CI 5.24–85.03, *p* < 0.0001; OR 11.6, 95% CI 4.51–29.57, *p* < 0.0001, respectively) (Fig. 1c).

### Neurological findings

The incidence and scores of nervous system manifestations were significantly higher in patients with HP than in those without HP in total AAV (*p* < 0.0001, *p* = 0.0008, respectively) and newly diagnosed AAV (*p* < 0.0001, *p* = 0.002, respectively), whereas they were not significantly different in relapsed AAV (*p* = 0.090, *p* = 0.114, respectively) (Table 2). Patients with HP had significantly higher frequencies of headache and cranial nerve palsy than those without HP in total AAV (*p* < 0.0001, *p* < 0.0001, respectively), newly diagnosed AAV (*p* < 0.0001, *p* = 0.0003, respectively), and relapsed AAV (*p* = 0.005, *p* = 0.003, respectively) (Table 4). The frequency of meningitis and spinal cord lesions was also significantly higher in patients with HP than in those without HP, although meningitis was not significantly different in newly diagnosed AAV. Conversely, mononeuritis multiplex was less frequently observed in patients with HP than in those without HP in total AAV (*p* = 0.009) and newly diagnosed AAV (*p* = 0.034).

**Table 4:** Comparison of neurological symptoms between patients with and without HP

	Total AAV			Newly diagnosed AAV			Relapsed AAV		
	HP (*n* = 30)	Non-HP (*n* = 633)	*p*-values	HP (*n* = 20)	Non-HP (*n* = 538)	*p*-values	HP (*n* = 10)	Non-HP (*n* = 95)	*p*-values
**Nervous system**, number (%)									
Headache	21 (70)	34 (5.7)	< 0.0001	16 (80)	29 (5.4)	< 0.0001	5 (50)	10 (11)	0.005
Meningitis	3 (10)	2 (0.3)	0.0007	1 (5)	1 (0.2)	0.071	2 (20)	1 (1.1)	0.023
Organic confusion	1 (3.3)	6 (0.9)	0.278	1 (5)	6 (1.1)	0.227	0	0	–
Seizures	0	4 (0.6)	1	0	2 (0.3)	1	0	2 (2.1)	0.999
Stroke	0	9 (1.4)	0.999	0	8 (1.5)	0.999	0	1 (1.1)	0.999
Spinal cord lesion	3 (10)	2 (0.3)	0.0008	1 (5)	1 (0.2)	0.007	2 (20)	1 (1.1)	0.023
Cranial nerve palsy	10 (33)	19 (3)	< 0.0001	5 (25)	15 (2.8)	0.0003	5 (50)	4 (4.2)	0.003
Sensory peripheral neuropathy	3 (10)	166 (26)	0.052	3 (15)	145 (27)	0.307	0	21 (22)	0.206
Mononeuritis multiplex	0	105 (17)	0.009	0	93 (17)	0.034	0	12 (13)	0.599

Statistical significance was set at a *p*-value < 0.05

*ANCA* antineutrophil cytoplasmic antibody, *AAV* ANCA-associated vasculitis, *HP* hypertrophic pachymeningitis

## Discussion

This study used the clinical information from a nationwide survey of AAV in Japan. We ultimately indicated the key findings that patients with HP had a higher prevalence of GPA and higher PR3-ANCA serum positivity than those without HP, and moreover, ENT and mucous membrane/eye manifestations were associated with HP development in AAV. Several single-center studies on ANCA-associated HP have been presented to date; however, it might be difficult to accurately determine the clinical characteristics contributing to HP development in AAV because of the limited number of patients analyzed at each institution. Considering the ethnic viewpoint of ANCA-associated HP, the majority of published studies have been reported from East Asian counties including Japan [8, 10, 15–20]. Consequently, this was the first study to assess the clinical characteristics of HP using the enrolled information from many Japanese patients with AAV, demonstrating the epidemiological findings, especially the frequency of HP and classification in AAV, as more reliable results than those in single-institutional studies. In addition, we also performed the analyses separately for newly diagnosed and relapsed AAV, elucidating consistent or different characteristics depending on the clinical stage of AAV in patients with HP. Our results showed an equal prevalence of GPA and MPA in patients with HP. Moreover, a higher classification of GPA was significant in patients with HP than in those without HP in the analyses of total and newly diagnosed AAV. It has also been suggested that patients with HP could be predominantly classified as having GPA in AAV [8, 10, 17–19], although the predominant prevalence of MPA has been found as an epidemiologic feature of AAV in Japan despite the dominance of GPA in European countries and the USA [22, 23]. The GPA classification could be dominantly found overall in patients with AAV who develop HP, even in Japan. Moreover, PR3-ANCA serum positivity in newly diagnosed AAV was significantly higher in patients with HP than in those without HP. Taken together, our results suggest that GPA with PR3-ANCA positivity may be predominantly observed in patients with HP in newly diagnosed AAV. PR3-ANCA serum positivity was found to be a risk factor for AAV relapse [24], but it might not be applicable to HP development in relapse. In contrast, HP related to MPO-ANCA could broadly develop in patients with both MPA and GPA throughout the course of AAV. In fact, the prevalence of HP was significantly lower in newly diagnosed AAV than in relapsed AAV, supporting the theory that CNS involvement could develop in the chronic phase of AAV [6, 7]. However, some cases of HP in AAV, whose initial diagnosis of MPA was changed to GPA, have been reported [8, 19, 25], suggesting that HP could be the initial episode of GPA.

Mucous membrane/eye manifestations were significantly more common in patients with HP than in those without HP; notably, sudden visual loss in this category [21] was a pivotal involvement in patients with HP. ENT manifestations were also significantly observed in patients with HP in total AAV and newly diagnosed AAV. In contrast, ENT dominance could not be significantly proved in relapsed AAV, suggesting that there was no significant difference in GPA classification between patients with and without HP because of increased GPA in patients without HP. However, conductive hearing loss in the ENT category, which is ascribable to middle ear impairment [21], was significantly involved in patients with HP even in relapsed AAV. Accordingly, mucous membrane/eye and ENT manifestations have been suggested as relevant clinical factors for HP development in AAV. Furthermore, sudden visual loss and conductive hearing loss were identified as principal involvements. Previous studies have suggested a relationship between otitis media and HP in AAV [8, 10, 17, 19, 26]. Only one study has revealed that visual impairment was more frequently observed in patients with HP than in those without HP in GPA [18]. In our study, both involvements were simultaneously demonstrated as significantly related manifestations in patients developing HP in AAV. Conversely, renal manifestations were relatively less involved in patients with HP, suggesting that the pathogenesis of renal damage may be less implicated in the development of HP. Considering the pathological aspects of AAV, granulomatosis with inflammatory cell infiltration has been more prominently exhibited in the upper and lower respiratory tracts, whereas necrotizing vasculitis, which widely affects small- to medium-sized vessels, typically indicates renal involvement [27, 28]. Although patients with HP rarely had mononeuritis multiplex, which is also ascribable to small- to medium-sized vasculitis [4, 29], the manifestations typically attributable to vasculitis might be little associated with HP development. It has been assumed that direct extension and/or transfer to the CNS from upper respiratory lesions, including granulomatosis inflammation, may be implicated in the mechanism for developing HP related to AAV [15, 16, 30]. Accordingly, our study also suggested that inflammatory lesions adjacent to the CNS, notably including eye and middle ear involvement, may be robustly implicated in HP development in AAV. In fact, it was demonstrated that HP, which was related to otitis media with AAV (OMAAV), was the most commonly localized in a cranial fossa [31].

This study also demonstrated that headache and cranial neuropathies were common neurological symptoms associated with HP in both newly diagnosed and relapsed AAV, consistent with previous reports [8, 10, 15–20]. In addition, our results indicated meningitis and spinal cord lesion as significant conditions in patients with HP. Cerebrospinal fluid (CSF) pleocytosis can be observed in ANCA-associated HP [10, 13, 17, 19]. However, the dura mater is outside the subdural space, which is anatomically separate from the CSF space [32, 33], suggesting that HP-mediated inflammation might extensively invade leptomeninges when pleocytosis can be observed. Moreover, meningitis may be involved in immune-mediated HP. Meanwhile, it was impossible to ascertain the types of spinal cord lesions that were analyzed because this study was performed using comprehensive items based on the BVAS. Therefore, localization of the thickened dura mater was uncertain, although HP can also develop in the spinal cord [10, 17, 34, 35].

There are other limitations in this study. First, some studies have suggested that mastoiditis, which was not evaluated in our study, is also frequently observed in patients with ANCA-related HP [10, 16, 19]. Nevertheless, this could develop as a result of the spreading effect of middle ear inflammation [36], suggesting that results are analogous to conductive hearing loss. Meanwhile, the frequency of mixed conductive-sensorineural hearing loss was found to be 9-fold higher than that of conductive hearing loss in patients with OMAAV [26]. However, it is difficult to differentiate conductive hearing loss from mixed conductive-sensorineural hearing loss in our study design based on BVAS. Second, cranial neuropathies were found to be significant manifestations of HP; however, it was impossible to identify specific types of impaired cranial nerves. Third, EGPA was ultimately not included in patients with HP, whereas some cases of HP in EGPA have been recently reported [17, 20, 37–39]. Although our study focused on the overview of HP in GPA or MPA, it is also necessary to elucidate the characteristics of HP in EGPA. Forth, brain MRI was not performed on all patients in the database of this study because the clinical information was retrospectively enrolled from the medical records. Therefore, it is necessary to establish the study design in which all patients perform brain MRI for more precise epidemiological analyses.

## Conclusions

GPA was classified in 50% of the patients with HP. The prevalence of GPA was significantly higher in patients with HP than in those without HP in total AAV and newly diagnosed AAV. Furthermore, patients with HP showed significantly higher PR3-ANCA serum positivity than those without HP in newly diagnosed AAV. Conversely, MPO-ANCA serum positivity was significantly higher in patients with HP than in those without HP in relapsed AAV. Taken together, GPA can be predominantly classified in patients with HP. Moreover, patients with HP classified as PR3-ANCA-positive GPA could be significantly observed in newly diagnosed AAV. HP with MPO-ANCA serum positivity could develop throughout the clinical course of AAV. ENT and mucous membrane/eye manifestations, notably sudden visual loss and conductive hearing loss, were relevantly identified in patients who develop HP in AAV. We expect that the accumulation of significant results determining clinical characteristics can be useful for predicting HP development in AAV. However, this study was performed using limited clinical information despite the first attempt at a multi-institutional survey. Accordingly, further investigation, by analyzing more detailed information from a larger number of patients, is required to elucidate the pathogenesis of HP in AAV.

## Supplementary Information

**Additional file 1: Table S1.** Comparison of demographic and clinical findings between newly diagnosed and relapsed AAV in patients with HP.

## Data Availability

The data for the analyses in this study are available on reasonable request.
