# Supplementary material for: Hypertrophic pachymeningitis in ANCA-associated vasculitis: a cross-sectional and multi-institutional study in Japan (J-CANVAS)
Source: Arthritis Res Ther. 2022 Aug 23;24:204. doi: 10.1186/s13075-022-02898-4 (PMC9396769; doi:10.1186/s13075-022-02898-4)
Supplement: Supplementary file 1 — Additional file 1: Table S1. Comparison of demographic and clinical findings between newly diagnosed and relapsed AAV in patients with HP. [file 13075_2022_2898_MOESM1_ESM.docx]

**Supplementary Table 1**

Comparison of demographic and clinical findings between newly diagnosed and relapsed AAV in patients with HP

|  |  |  | **Newly diagnosed** | **Relapsed** | ***P*-values** |
| --- | --- | --- | --- | --- | --- |
| **HP in total AAV** | | |  |  |  |
|  | Number (%) | | 20 (3.6) | 10 (9.5) | 0.017 |
| **Characteristics** | | |  |  |  |
|  | Age, year | | 70.8±8.5 | 71.9±7.5 | 0.808 |
|  | Sex, male/female, number (%) | | 12/8 | 6/4 | 0.999 |
| **AAV classification**, number (%) | | |  |  |  |
|  | GPA | | 10 (50) | 5 (50) | 0.999 |
|  | MPA | | 10 (50) | 5 (50) | 0.999 |
|  | EGPA | | 0 | 0 | ― |
| **Type of ANCA**, number (%) | | |  |  |  |
|  | PR3 | | 5 (25) | 1 (10) | 0.633 |
|  | MPO | | 14 (70) | 9 (90) | 0.371 |
|  | Seronegative | | 1 (5) | 0 | 1 |
| **BVAS, total** | | |  |  |  |
|  |  | Total score | 14.0±5.40 | 12.3±5.98 | 0.452 |
| **Manifestation** | | |  |  |  |
|  | **Incidence**, number (%) | |  |  |  |
|  |  | General | 11 (55) | 2 (20) | 0.119 |
|  |  | Cutaneous | 2 (10) | 0 | 0.540 |
|  |  | Mucous membranes/eyes | 6 (30) | 5 (50) | 0.425 |
|  |  | ENT | 13 (65) | 3 (30) | 0.122 |
|  |  | Chest | 7 (35) | 3 (30) | 0.999 |
|  |  | Cardiovascular | 0 | 0 | ― |
|  |  | Abdominal | 0 | 0 | ― |
|  |  | Renal | 10 (50) | 2 (20) | 0.235 |
|  |  | Nervous system | 18 (90) | 7 (70) | 0.300 |
|  | **Score*** | |  |  |  |
|  |  | General | 1.35±1.31 | 0.40±0.84 | 0.051 |
|  |  | Cutaneous | 0.20±0.62 | 0 | 0.334 |
|  |  | Mucous membranes/eyes | 1.05±1.93 | 2.60±2.99 | 0.183 |
|  |  | ENT | 3.05±2.56 | 1.50±2.55 | 0.137 |
|  |  | Chest | 1.45±2.16 | 1.60±2.63 | 0.979 |
|  |  | Cardiovascular | 0 | 0 | ― |
|  |  | Abdominal | 0 | 0 | ― |
|  |  | Renal | 3.60±4.38 | 2.20±4.66 | 0.242 |
|  |  | Nervous system | 3.30±3.16 | 4.00±3.46 | 0.982 |
| **Laboratory data** | | |  |  |  |
|  | White blood cells, /μL | | 10510±4628 | 7834±2069 | 0.244 |
|  | Neutrophils, /μL | | 8635±4889 | 6842±2102 | 0.629 |
|  | Lymphocytes, /μL | | 1269±632 | 587±310 | 0.008 |
|  | C-reactive protein, mg/dL | | 7.35±5.70 | 3.97±4.86 | 0.104 |
|  | Serum creatinine, mg/dL | | 0.78±0.33 | 0.82±0.44 | 0.929 |
|  | eGFR | | 75.8±23.0 | 76.5±29.5 | 0.826 |

ANCA, antineutrophil cytoplasmic antibody; AAV, ANCA-associated vasculitis; BVAS, Birmingham Vasculitis Activity Score version 3; EGPA, eosinophilic granulomatosis with polyangiitis; ENT, eye, nose and throat; eGFR, estimated glomerular filtration rate; GPA, granulomatosis with polyangiitis; MPA, microscopic polyangiitis; HP, hypertrophic pachymeningitis; Values are expressed as mean ± SD. *Scored for each manifestation item based on the BVAS [1]. Statistical significance was set at a *P*-value < 0.05.

1. Mukhtyar C, Lee R, Brown D, Carruthers D, Dasgupta B, Dubey S, et al. Modification and validation of the Birmingham Vasculitis Activity Score (version 3). Ann Rheum Dis 2009;68(12):1827-32.
